# Insulin-like growth factor 1 injection changes gene expression related to amino acid transporting, complement and coagulation cascades in the stomach of tilapia revealed by RNA-seq

**DOI:** 10.3389/fimmu.2022.959717

**Published:** 2022-08-09

**Authors:** Huan Zhong, Chenyi Lou, Bingxin Ren, Yi Zhou

**Affiliations:** ^1^ Hunan Research Center of Engineering Technology for Utilization of Distinctive Aquatic Resource, College of Animal Science and Technology, Hunan Agricultural University, Changsha, China; ^2^ State Key Laboratory of Developmental Biology of Freshwater Fish, Life Science College, Hunan Normal University, Changsha, China

**Keywords:** complement and coagulation systems, IGF-1, transcriptome, tilapia, stomach

## Abstract

Insulin-like growth factor 1 (IGF-1) is a key hormone that regulates fish growth. It acts on a variety of organs and regulates multiple signaling pathways. In order to explore the specific effects of IGF-1 on fish nutrient absorption, immune system, and other functions, the present study investigated the transcriptional changes of stomachs in tilapia by IGF injection. The tilapias were divided into two groups which were injected with saline (C group) and IGF-1 (2 μg/g body weight) (I group), respectively. After three times injections, the stomachs from the tested tilapias were collected 7 days post the first injection and the transcriptomes were sequenced by Illumina HiSeqTM 2000 platform. The results showed that a total of 155 DEGs were identified between C and I groups. By gene ontology (GO) enrichment analysis, two GO terms related to absorption function were enriched including organic acid transport, and amino acid transport which contained 6 functional DEGs. The Kyoto Encyclopedia of Genes and Genomes (KEGG) enrichment analysis suggested that *Staphylococcus aureus* infection, as well as complement and coagulation cascades pathways were enriched and contained 6 DEGs. Taken together, the present study indicated that IGF-1 injection altered gene expression related to amino acid transporting, complement and coagulation cascades which provides a promise immunopotentiation therapy by IGF-1 in digestive tract of tilapia.

## Introduction

Growth of fish is one of the most concerned characteristics in aquaculture, which is mainly controlled by the key hormones in growth hormone/insulin like growth factor-1 (GH/IGF-1) axis (1, 2). As a key hormone in GH/IGF-1 axis, IGF-1 has other functions besides regulating growth (3). Several studies have shown that IGF-1 plays an important role in immune system of teleost, which participates in proliferation, differentiation and function of immune cells (4, 5). Moreover, IGF-1 can also regulate the osmoregulation of fish (6). These studies suggest that IGF-1 can regulate multiple physiological processes. Recent studies indicated that the specific receptor of IGF-1 (IGF-1R) had high expression in fish stomachs suggesting the potential regulatory function of IGF-1 such as altering the expression of genes in stomachs (7, 8). Meanwhile, IGF-1 is regulated by many hormone pathways, including ghrelin/growth hormone secretagogue receptor (GHS-R) axis (9). These clues suggest that the network composed of GH/IGF-1 axis and ghrelin-GHS-R axis plays a key role in gastric transcription regulation (10). Thus, clarifying the regulation of IGF-1 on gastric transcription level is helpful to the comprehensive understanding of IGF-1 and promotes the study of hormone-regulating digestion and absorption.

Endocrine regulation plays an important role in maintaining normal physiological activities of fish *via* humoral regulation generally. According to previous studies, the endocrine regulates several biological processes such as growth, reproduction and immunity in fishes (11, 12). Due to these key functions, the application of endocrine provides great economic benefits for aquaculture (13, 14). Commonly, endocrine regulation is executed by secretion of hormones from secretory cells or tissues, which are transported to target cells through body fluids (15). Gastrointestinal tract has been regarded as one of the main secretory tissues (16, 17). Interestingly, stomach is one of the main secretory tissues, but not all fishes have it. For example, tilapia has stomach but crucian carp and common carp are stomachless fishes (18). For fish, stomach not only helps it digest food better, but connects endocrine with nervous system through releasing hormones. One of the important hormones secreted by stomach is ghrelin, which can regulate the expression of GH/IGF-1 axis to regulate the growth of fish (19). Recent studies have been reported that stomachs could also be under control by feedback regulation of several hormones (20, 21). However, the regulation of IGF-1 on gene expression in fish stomach has not been studied systematically. This situation hinders the understanding of the multi-functional regulation of key growth hormones.

Tilapia is one of the common farmed fishes in south China, whose cultivation has provided significant economic benefits. It is of great significance to study the feeding utilization and immunomodulatory function of tilapia (22, 23). Stomach, one of the main digestive organs, is regulated by multiple hormones, including IGF-1. However, the specific regulatory mechanism of IGF-1 is still unknown in tilapia. Clarifying the regulation of IGF-1 on the stomach will not only help us to have a better understanding of function of stomach, but also help us to understand the multi-function of IGF-1. To find out its specific regulatory effect, we studied the impact of IGF-1 on the transcriptome of tilapia stomach. In the present research, the transcriptomes of stomachs were sequenced from tilapias injected with saline or IGF-1. The data were processed and differentially expressed genes (DEGs) were identified from the control and IGF-1 groups. The function of DEGs was defined by gene ontology (GO) terms and Kyoto Encyclopedia of Genes and Genomes (KEGG) pathways. Real-time fluorescent quantitative PCR (qPCR) of the differentially expressed genes was performed to confirm the accuracy of RNA-seq. These results would aid to deduce the specific regulation of IGF-1 on fish stomach.

## Materials and methods

### Fish and injection

**Figure 1:**
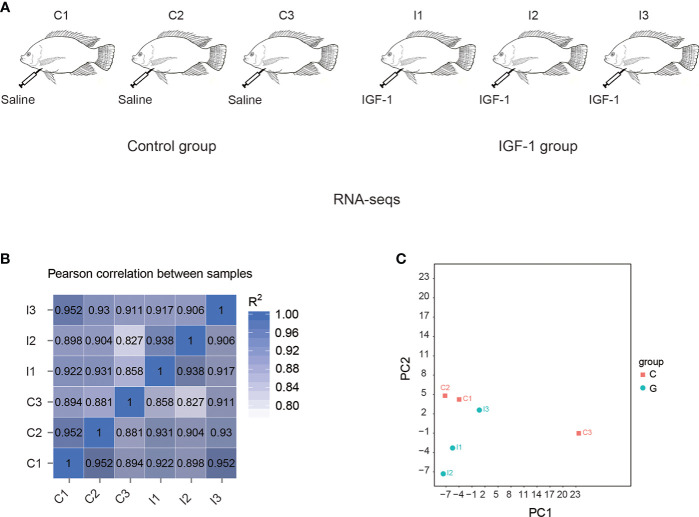
RNA-seq of stomachs in tilapia by IGF-1 injection. **(A)** The experimental group was injected with IGF-1 (I group), and the control group was injected with normal saline (C group). **(B)** Correlation matrix among the samples. **(C)** Principal component analysis of the transcriptomes in the stomach of I group and C group.

Nile tilapia (*Oreochromis niloticus*) used in the present study were obtained from Guangxi Academy of Fishery Sciences (Nanning, Guangxi). Ten adult individuals (the bodyweight was 0.38 ± 0.05 kg) were randomly selected and maintained in two separated tanks (n=5) (1 m^3^ × 1 m^3^ × 1.5 m^3^) with a continuous flow system under 12 h light/12 h dark photoperiod. The water temperature, pH and dissolved oxygen were 25-28°C, 7.2 and 6.50-8.00 mg/L, respectively. All the experiments were approved by Ethics Committees of Hunan Agricultural University (Changsha, China). The individuals from two tanks were assigned to the two groups including control group (C group) and IGF-1 group (I group). The fish in C group were injected with saline and the fish in I group were injected with 2 μg/g body weight IGF-1 (Sigma, USA) at day 1, day 3 and day 5 of the experimental trial according to the previous report (24). All the fish in the present study were intraperitoneally injected. The stomachs of the fish were collected at the day 7 post injection. For each group, 3 individuals were randomly collected for the RNA-seq (**Figure 1A**).

### RNA extraction and quality evaluation

Total RNAs of samples were extracted by TRIzol reagent (Takara, Japan) according to the manufacturer’s instruction. The integrity and concentration of the extract were assayed using 1% agarose gel electrophoresis and NanoDrop 2000 spectrophotometer (Thermo Scientific, USA), respectively. Subsequently, the evaluation of RNA quality was accomplished by Aligent 2100 (Aligent, USA).

### cDNA libraires construction and RNA-seq

The mRNA was obtained from total RNA by oligo (dT)-attached magnetic beads and then fragmented to 100 bp to 500 bp fragments. Using NEB Next Ultra RNA Library Prep Kit (Illumina, USA) and random hexamer primers, the cDNA of these fragments was synthesized. Subsequently, PCR was used to amplify the cDNA and the products were purified by agarose gel electrophoresis. Eventually, the amplificated cDNA was sequenced using Illumina HiSeqTM 2000 platform (Illumina, USA).

### Reads processing and function annotation

To get clean reads, raw reads were processed by in-house perl scripts, which could remove adaptor sequences and low-quality reads whose number of bases with low sequencing quality of reads accounts for a high proportion. The genome of Nile tilapia (https://asia.ensembl.org/Oreochromis_niloticus/Info/Index) was used as a reference database and clean reads were mapped to it by RSEM software (25). Subsequently, GO (http://geneontology.org/) analysis was carried out to ensure molecular function, cellular component and biological process of genes and KEGG (https://www.genome.jp/kegg/) was used to know genes functions which focused on biochemical pathways.

### Identification of differentially expressed genes

Fragments per kilobase of exon model per million mapped reads (FPKM) of each gene were calculated to analyze gene expression levels. Subsequently, DESeq R package was used to identify DEGs (26). Genes with adjusted p-value ≤ 0.05 and |log2 fold change| ≥ 1 were regarded as significant differential expression.

### GO and KEGG enrichment analysis

To understand which metabolisms in organisms all DEGs are associated with, GO and KEGG enrichment analysis were implemented. The GOseq R package based on Wallenius non-central hyper-geometric distribution was used to analyze GO enrichment (27). When adjusted p-value ≤ 0.05, genes were considered as differentially expressed. Using KOBAS software (KOBAS, Surrey, UK), differential expression genes (p-value ≤ 0.05) in KEGG pathways were enriched (28).

### qPCR

The total RNA extracted and purified was used to reverse transcription. By PrimeScript™ RT reagent Kit (Takara), the qualified RNAs (1 μg for each sample) were transformed to cDNA. gDNA Eraser (Takara) with DNase I was applied to eliminate genomic DNAs. The synthesized cDNAs were qualified using β-actin primers.

Subsequently, the RT-qPCR of DEGs were designed by Primer Premier 5.0 (Premier Biosoft International, USA) (**Table S1**). The qPCR which used PowerTrack SYBR Green Master Mix (ABI, USA) as fluorescent dye was performed on the QuantStudio™ 3 Real-Time PCR System (ABI, USA). The amplification system was as follows: 1 μl of cDNA (1:40 dilution), 0.5 μl of forward and reverse primers separately, 5 μl of PowerTrack SYBR Green Master Mix, and 3 μl of H_2_O. The system cycled 40 times, and each time it was 50°C for 2 min, 95°C for 10 min, 95°C for 15 s and 60°C for 45 s. Finally, the melting curve analysis was to ensure the specificity of PCR. In this study, internal reference was β-actin gene. The 2^−ΔΔCt^ method was used to calculate relative expression (29) and t-test by SPSS v17.0 (SPSS, Chicago, IL, USA) was used to analyze significant differences.

## Results

### Analysis of RNA-seq

In this study, six samples including C1, C2, C3, I1, I2 and I3 were analyzed, generating 297.37 million raw reads. For the samples, each group averaged about 49.56 million raw reads. All the reads were available on NCBI SRA database (https://www.ncbi.nlm.nih.gov/bioproject/?term=PRJNA846796). After removing adaptor sequences and low-quality reads, about 49.21 million clean reads of each sample were generated (**Table S2**). Among them, an average of 89.59% clean reads in each sample mapped to the genome (**Table S3**).

By correlation analysis (**Figure 1B**), high similarity in parallel samples could be found. The correlation among C1, C2 and C3 was from 0.881 to 0.952, while for I1, I2 and I3, it was from 0.906 to 0.938. Principal component analysis (PCA) demonstrated differences of expression between the transcriptomes of C group and I group (**Figure 1C**).

### GO and KEGG enrichment analysis of DEGs

**Figure 2:**
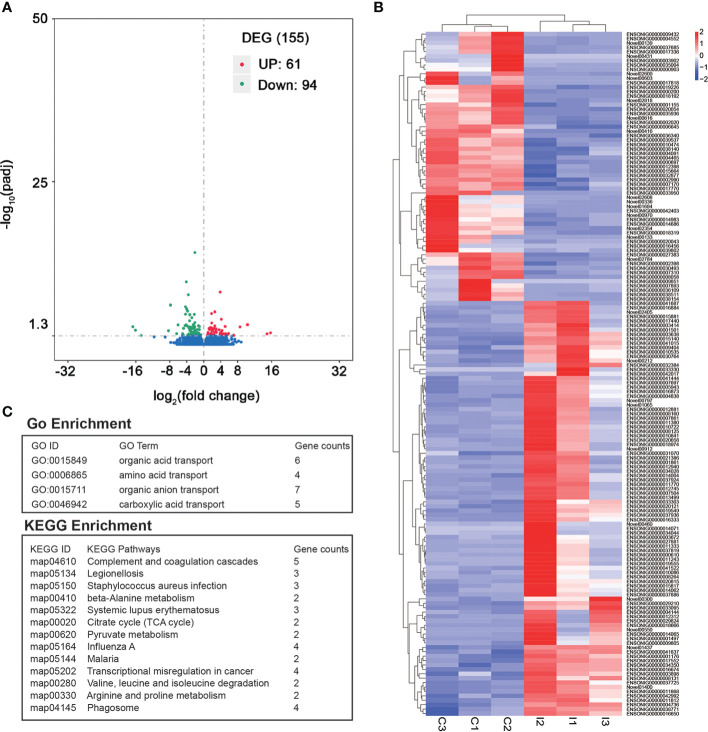
Identification of DEGs between IGF-1 injection group and control group. **(A)** The volcano plot of DEGs. “UP” showed the genes had higher expression in C group compared to I group. “DOWN” showed the genes had higher expression in I group compared to C group. **(B)** Heatmap of all the DEGs. **(C)** GO enrichment and KEGG enrichment analysis of DEGs.

A total of 155 DEGs were identified between C and I group. Among these DEGs, the expressions of 61 genes were significantly higher in C group than that of I group (**Table S4**). In contrast, 94 genes had lower expression in C group than that of I group (**Figure 2A, B**). The GO enrichment analysis of the DEGs suggested that 4 GO terms were significantly enriched (**Table S5**). Among these enriched GO terms, organic acid transport and amino acid transport are associated with growth and development (**Figure 2C**). The result showed 13 KEGG pathways were significantly enriched (**Table S6**). Among these pathways, complement and coagulation cascades and *Staphylococcus aureus* infection were related to disease resistance (**Figure 2C**).

### IGF-1 injection changes genes related to amino acid transporting

**Figure 3:**
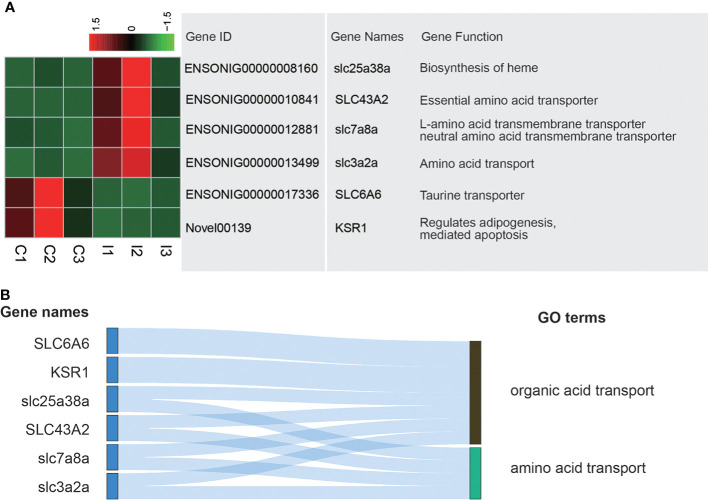
Analysis of DEGs related to amino acid transporting and organic acid transporting terms. **(A)** Heatmap and gene annotation of DEGs in amino acid transporting and organic acid transporting GO terms. **(B)** Attribution of DEGs in amino acid transporting and organic acid transporting GO terms.

Because the stomach had the functions of digestion and absorption and was the main organ to absorb a variety of amino acids and organic acids, the organic acid transport terms and amino acid transport terms were the focus of the study. A total of 6 genes in these terms were differently expressed. Among them, gene expression of slc25a38a, SLC43A2, slc7a8a and slc3a2a in I group was higher than that of C group. On the contrary, gene expression of SLC6A6 and KSR1 was higher in C group (**Figure 3A**). Among these genes, SLC6A6 and KSR1 were related to organic acid transport, while slc25a38a, SLC43A2, slc7a8a and slc3a2a were associated with both organic acid transport and amino acid transport (**Figure 3B**).

### IGF-1 injection affects genes related to complement and coagulation cascades

**Figure 4:**
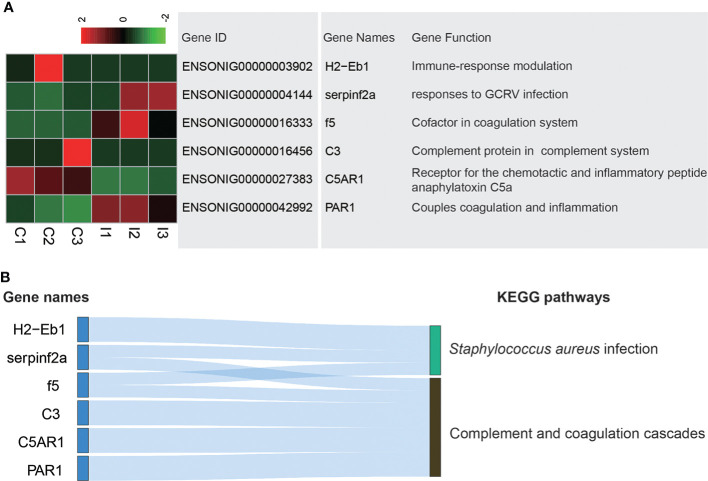
Analysis of DEGs related to Complement and coagulation cascades and *Staphylococcus aureus* infection pathways. **(A)** Heatmap and gene annotation of DEGs in complement and coagulation cascades and *Staphylococcus aureus* infection pathways. **(B)** Attribution of DEGs in complement and coagulation cascades and Staphylococcus aureus infection pathways.

The stomach has immune function, which can prevent the invasion of pathogenic microorganisms and foreign substances. Complement and coagulation cascades pathway is an important in defense against pathogen invasion and *Staphylococcus aureus* infection pathway is carried out to prevent infection of *Staphylococcus aureus*. Six DEGs existed in these pathways. H2−Eb1, C3 and C5AR1 expressed higher in C group, while serpinf2a, f5 and PAR1 expressed higher in I group (**Figure 4A**). H2−Eb1 played a role in *Staphylococcus aureus* infection. C3, C5AR1 and PAR1 were related to complement and coagulation cascades. Serpinf2a and f5 were associated with both *Staphylococcus aureus* infection and complement and coagulation cascades (**Figure 4B**).

### RT-qPCR of immune related genes affected by IGF-1 injection in stomach

**Figure 5:**
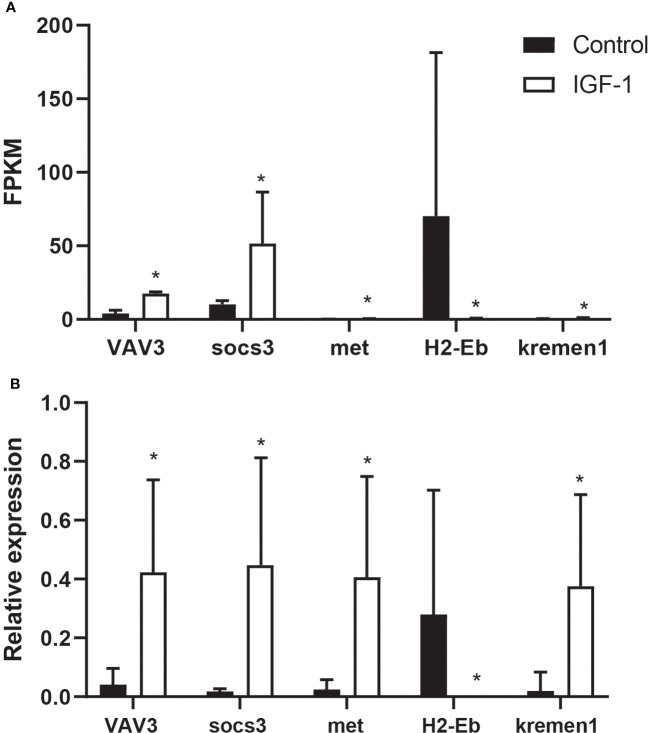
The comparison of FPKM and RT-qPCR of VAV3, socs3, met, H2-Eb and kremen1 in IGF-1 injection and control group. **(A)** The FPKM of VAV3, socs3, met, H2-Eb and kremen1. * showed the significant difference (adjusted p-value ≤ 0.05 and |log2 fold change| ≥ 1). **(B)** RT-qPCR results of VAV3, socs3, met, H2-Eb and kremen1. * showed the significant difference (P ≤ 0.05).

In order to verify the accuracy of RNA-seq, this study used qPCR to detect gene expression. VAV3, socs3, met, H2-Eb and kremen1 were detected, all of which were related to immunity. The expression level of VAV3, socs3, met and kremen1 increased in I group, while H2-Eb expressed lower in I group than that in C group (**Figure 5**). Comparing qPCR with RNA-seq, it was found that results were consistent, which proved the accuracy of RNA-seq.

## Discussion

In this study, the transcriptome of the stomach of tilapia injected with IGF-1 was measured and compared with the control group injected with saline, to explore the effect of IGF-1 on the stomach of tilapia and understand the regulatory mechanism of IGF-1. Through this study, we discovered that IGF-1 affected the expression of multiple genes in the stomach. The digestion and absorption function as well as immune function of the stomach were the focus of the research, thus, the changes of these related functional genes after injection of IGF-1 were further analyzed. The mechanism of these gene changes was of significance for clarifying the regulation and effect of IGF-1 on the stomach. The research provided a new insight that injection IGF-1 can promote absorption and enhance their disease resistance in the process of production and breeding.

The amino acid transport and organic acid transport capacities of the stomach were important components of its absorption. The study found that six genes related to amino acid transport and organic acid transport were significantly different after injection of IGF-1. Among them, the SLC6A6 and KSR1, which are related to the organic acids transport, have higher expression in the C group. However, slc25a38a, SLC43A2, slc7a8a and slc3a2a related to both amino acids transport and organic acids transport were higher in I group compared to C group. These genes belong to the solute carrier (SLC) superfamily (30), which are proteins that promote the uptake of small molecules and are highly expressed in the digestive system (31). From expression changes of genes in SLC superfamily, IGF-1 may affect gastric digestion and absorption (32). The analysis of up-regulated genes number in I group and their functions indicated that the IGF-1 injection may increase the absorption capacity. This result showed that IGF-1 may improve the absorption function of the stomach which was similar to the studies in mammalian (33, 34).

IGF-1 altered the expression of immune-related genes (35, 36). *Staphylococcus aureus* infection was a common fish disease that caused huge losses in fishery production (37). The ability to combat bacterial infections in the immune response is critical for the healthy growth of fish. The 3 DEGs of H2-Eb1, serpinf2a and f5 associated with *Staphylococcus aureus* infection suggested that IGF-1 may promote disease resistance. In bacterial infection of rainbow trout (*Oncorhynchus mykiss*), a strong correlation between IGFBP-1A1 and IGFBP-6A2 in the GH/IGF axis and proinflammatory cytokine genes was found, which suggested a relationship between IGF-1 and the immune response against bacteria (38). Complement and coagulation cascades is an important pathway in the immune response (39). Transcriptome sequencing of large yellow croaker (*Larimichthys crocea*) with *Cryptocaryon irritans* infection showed that the abundant DEGs were found in complement and coagulation cascades pathway (40). In this experiment, among the 5 DEGs related to complement and coagulation cascades pathway, only C3 and C5AR1 expressed highly in C group. Both C3 and C5AR1 belong to the complement cascade, which mainly mediate immune and inflammatory responses (41). The remaining 3 DEGs that were highly expressed in I group were involved in the coagulation cascade and the interaction of the coagulation cascade and the complement cascade (42). Based on these results, we proposed that IGF-1 had immunomodulatory function in tilapia stomach.

In summary, in the present study, by analyzing the changes in the transcription level after injection of IGF-1, the genes that participated in the pathways closely related to gastric absorption and immune function were identified. The present study provided actionable clues like injecting IGF-1 or increasing the expression of endogenous IGF-1 for promotion of nutrient utilization and disease resistance of tilapia in aquaculture.

## Data availability statement

The datasets presented in this study can be found in online repositories. The names of the repository/repositories and accession number(s) can be found below: NCBI (SRA), accession ID: PRJNA846796.

## Ethics statement

The animal study was reviewed and approved by Ethics Committees of Hunan Agricultural University (Changsha, China).

## Author contributions

HZ designed the experiment and wrote the manuscript. CL and BR performed the data analysis and the experiment. YZ reviewed and revised the manuscript. All the authors approved the manuscript for publication.

## Funding

This study was supported by National Natural Science Foundation of China (31760756) and Hunan Provincial Natural Science Foundation of China (2021JJ20033).

## Conflict of interest

The authors declare that the research was conducted in the absence of any commercial or financial relationships that could be construed as a potential conflict of interest.

## Publisher’s note

All claims expressed in this article are solely those of the authors and do not necessarily represent those of their affiliated organizations, or those of the publisher, the editors and the reviewers. Any product that may be evaluated in this article, or claim that may be made by its manufacturer, is not guaranteed or endorsed by the publisher.
